# Comparison of Standard and New Iron Status Biomarkers: A Prospective Cohort Study in Sepsis Patients

**DOI:** 10.3390/healthcare11070995

**Published:** 2023-03-30

**Authors:** Piotr F. Czempik, Agnieszka Wiórek

**Affiliations:** 1 Department of Anaesthesiology and Intensive Care, Faculty of Medical Sciences in Katowice, Medical University of Silesia, 40-055 Katowice, Poland; 2 Transfusion Committee, University Clinical Centre of Medical University of Silesia, 40-055 Katowice, Poland

**Keywords:** ferritin, hepcidin, iron, reticulocyte hemoglobin, sepsis, septic shock, transferrin, transferrin saturation

## Abstract

Both iron deficiency (ID) and iron overload can have negative effects on the risk and course of infection. Therefore, the ability to accurately assess iron status in these patients is of the utmost importance. Systemic inflammation in sepsis patients affects the results of standard iron biomarkers and makes accurate diagnosis of ID problematic. The aim of our study was to analyze the association between widely available standard iron biomarkers and selected new iron biomarkers in various iron status subgroups among sepsis patients. Consecutive patients diagnosed with sepsis or septic shock and procalcitonin concentration > 0.5 ng/mL were enrolled. The following iron biomarkers were determined: iron, ferritin, transferrin, transferrin saturation, reticulocyte (Ret) number and percentage, Ret hemoglobin equivalent, Ret fluorescence subpopulations, and hepcidin concentration. The study group comprised 90 study subjects. There were 42 (47%) patients with normal iron status, 6 (6%) with ID without anemia, and 42 (47%) with ID anemia. No meaningful correlation exists between standard and new iron biomarkers in various iron status subgroups among sepsis patients. Therefore, standard iron biomarkers cannot be used to diagnose ID in this cohort.

## 1. Introduction

Iron is a trace element necessary for numerous physiological processes. Its role in hemoglobin (Hb) synthesis and immune function is especially important in the context of sepsis. Hemoglobin is required for oxygen transport and delivery to the cells. Adequate immune function is necessary to prevent and fight infection. Iron in macrophages regulates the production of cytokines [1]. Iron concentration is tightly controlled, and both iron deficiency (ID) and iron excess are deleterious. Newly absorbed iron binds to plasma transferrin and is distributed around the body, mainly to the bone marrow, having particularly high requirements associated with erythropoiesis. Macrophages phagocytose senescent red blood cells (RBCs) with iron being released from catabolized Hb and reentering the circulation [2]. Iron deficiency may be present before anemia develops and may already, at this stage, be deleterious. Both ID and iron overload can have particularly adverse effects on the risk and course of infection, which suggests a U-shaped relationship between iron status and infection risk [3]. During infection, iron may be sequestrated, which constitutes a defense mechanism, as bacteria require iron for growth. Harmful effects of iron in the setting of infection have been linked to elevated serum ferritin and non–transferrin-bound iron rather than iron itself [4].

It is vital to accurately diagnose ID in patients at risk of infection or with ongoing infection. However, iron metabolism in sepsis patients undergoes significant changes leading to iron sequestration. Iron sequestration is characteristic of anemia of inflammation (AI). Sepsis patients, apart from having AI, may also be iron-deficient, so that they may develop a mixed type of anemia–AI combined with iron deficiency anemia (IDA). This fact makes the diagnosis of ID in sepsis patients difficult.

Systemic inflammation in sepsis patients affects the results of standard iron tests, complicating the diagnosis of ID [5]. There are relatively new laboratory parameters that can be used for the precise diagnosis of iron deficiency without anemia (IDWA) and IDA, accompanying AI in sepsis patients. A reticulocyte (Ret) parameter called reticulocyte hemoglobin equivalent (Ret-He) has been researched extensively in recent years. Reticulocyte Hb equivalent has been used as a marker of ID in various patient populations. This parameter reflects iron availability during the synthesis of Rets, i.e., over the last 3–4 days, and may be used to diagnose IDWA/IDA in sepsis patients. The critical advantage of this test is its wide availability and minimization of blood lost for IDWA/IDA diagnostics, as Hb and erythrocyte/Ret indices are determined from a single complete blood count (CBC) EDTA test tube (2 mL test tube in the authors’ institution). Hepcidin (Hepc) is another parameter that has shown promise in distinguishing pure AI from a mixed type of anemia (AI+IDA) in sepsis patients. Hepcidin is the master regulator hormone of iron metabolism, which regulates iron absorption in enterocytes and the release of iron from the reticuloendothelial system. Analytic methods for Hepc are not widely available in the hospital setting. Additionally, other biomarkers can be used for ID diagnostics; however, their availability may differ.

As the classic iron biomarkers are widely available and may be the only parameters used for iron status assessment at a particular institution, the aim of our study was to analyze the association between these tests and new laboratory biomarkers in various iron status sepsis patients.

## 2. Materials and Methods

### 2.1. Type of the Study

This prospective clinical study was conducted between September 2021 and August 2022. The study was conducted in a 10-bed medical-surgical intensive care unit (ICU) at a tertiary care academic medical center in Poland. The study was registered retrospectively at clinicaltrials.gov (NCT05217836).

### 2.2. Study Subjects

Patients with sepsis or septic shock and a procalcitonin concentration >0.5 ng/mL were enrolled in the study. For the diagnosis of sepsis and septic shock, the third international definition and appropriate diagnostic criteria were used [6]. The aforementioned cut-off for PCT was chosen because, at this concentration, systemic infection is likely and systemic inflammation is pronounced [7]. Moreover, diagnosis of sepsis or septic shock may be difficult in critically ill patients commonly presenting signs of systemic inflammation. Hence, Japanese sepsis guidelines advise using PCT to improve the accuracy of sepsis diagnosis [8]. Common anatomical sites of infection were: pulmonary (n = 32/36%), abdominal (n = 21/23%), urinary (n = 19/21%), biliary (n = 7/8%), meningitis (n = 2/2%), Central Line-Associated Bloodstream Infection (CLABSI; n = 2/2%), other or unspecified (n = 7/8%). The exclusion criteria included conditions that could have an impact on the diagnosis of anemia or iron deficiency: active bleeding (decrease in Hb concentration), RBC transfusion in the last three months (impact on hematological parameters), use of oral or intravenous iron preparations in the previous three months (impact on iron biomarkers), pregnancy (different cut-off value for the diagnosis of anemia), mean cell volume (MCV) above the upper reference value (falsely increases Ret-He), and thalassemia (falsely decreases Ret-He). We divided study subjects into three groups: normal iron status (NIS), iron deficiency without anemia (IDWA), and iron deficiency anemia (IDA). Diagnosis of anemia was based on the World Health Organization (WHO) diagnostic criteria: Hb concentration < 120 g/L in non-pregnant women and <130 g/L in men [9]. Iron status was based on the results of Ret-He and Hb. The diagnostic criteria for the aforementioned patient subgroups are given in Table 1. 

### 2.3. Laboratory Determinations

All blood samples were taken on the ICU admission. In all study subjects baseline general laboratory parameters were determined for PCT, C-reactive protein (CRP), alanine aminotransferase, aspartate aminotransferase, total bilirubin, creatinine, and blood urea nitrogen. The following iron biomarkers were determined: iron, ferritin, transferrin, CBC, Ret number and percentage, Ret-He, Ret fluorescence subpopulations, and hepcidin concentration. Transferrin saturation was calculated using the following formula: iron [µg/dL]/transferrin [mg/dL] × 71.6. The blood for determination of CBC and Rets analysis was collected in a single 2 mL EDTA test tube (BD Vacutainer, Becton Dickinson, UK), and the test was performed using a standard laboratory hematology analyzer (XN-1000, Sysmex, Kobe, Japan). The advantage of Ret-He is that it allows for early identification of IDWA/IDA, as this parameter changes well before RBC indices become abnormal and reflects the current bone marrow iron availability [10]. Iron deficiency was diagnosed when Hb concentration was normal (≥120 g/L in women; ≥130 g/L in men), but Ret-He was below the lower limit of the reference range (i.e., <30.2 pg). We used the manufacturer’s reference range for Ret-He (30.2–36.2 pg) because various cut-off values for Ret-He were proposed in different patient populations: 25 pg [11], 28 pg [12], 29 pg [13], and 30 pg [14]. Moreover, these cut-off values were based on standard iron serum markers, for example, transferrin saturation [15,16]. These cut-off values may not be appropriate for sepsis patients. Moreover, in our study, we distinguished between IDWA and IDA. Iron deficiency anemia was diagnosed when the Hb concentration was below the reference range (i.e., <120 g/L in women; <130 g/L in men) and Ret-He was <30.2 pg. Blood for hepcidin determination was centrifuged, frozen, and stored at −80 °C for batch determination. Enzyme-Linked Immunosorbent Assay (ELISA) Kit for Hepcidin (Hepc) was used. All Hepc determinations were performed according to the instructions provided by the manufacturer (Cloud-Clone Corporation, Katy, TX, USA) by an experienced laboratory technician.

### 2.4. Statistical Analysis

Statistical analysis was performed using MedCalc v.18 software (MedCalc Software, Ostend, Belgium). Quantitative variables were depicted using medians and interquartile ranges (IQR, i.e., 25pc–75pc). The Shapiro-Wilk test was used to verify their distributions. Qualitative variables were described with frequencies and percentages. Between-group differences for continuous variables with normal distribution were assessed with ANOVA one-way analysis of variance, and continuous variables with non-normal distribution were evaluated using the Kruskal–Wallis test. For variables with statistically significant initial between-group differences, a post-hoc analysis was performed to determine between which subgroups the values of the variables differed significantly. The Chi-squared test was applied for categorical variables. Receiver Operating Characteristic (ROC) curves were drawn, and areas under ROC curves (AUROC) were calculated to determine the predictive value of studied parameters and iron deficiency. An analysis of ROC curves was also performed to statistically assess the optimal cut-off values for outcome prediction. Correlations were assessed using Spearman’s rank coefficient. The correlation coefficient value was interpreted from the published literature [17]. A *p*-value < 0.05 was considered significant.

## 3. Results

The study group comprised 90 study subjects. There were 42 (46.7%) patients with NIS, 6 (6.6%) with IDWA, and 42 (46.7%) with IDA. The median age in NIS, IDWA, and IDA subgroups was 63 (IQR 48–68), 61 (IQR 46–71), and 69 (IQR 61–77) years, respectively. The number of men in these subgroups was 26 (62%), 3 (50%), and 22 (52%), respectively.

A comparison of general laboratory parameters in the iron status subgroups is presented in Table 2.

There were statistically significant differences between all iron status subgroups for RBC, Hb, and hematocrit (*p* for all comparisons < 0.001). There were no differences between the iron status subgroups for MCV, MCH, MCHC, RDW-SD, and RDW-CV. Therefore, these parameters are not useful for the diagnosis of IDWA or IDA. 

A comparison of iron status laboratory parameters in the iron status study subgroups is presented in Table 3.

There were no statistically significant differences between iron status subgroups for standard iron tests, iron, transferrin, TSAT, and ferritin. There were statistically significant differences between NIS and IDA for percentage and number of Rets. Both these parameters were within the reference range in sepsis patients with IDA. The Ret percentage was at the upper limit (1.44%), and the Ret number was at the lower limit (0.05 × 10^6^/µL). The only parameter that was significantly different between all subgroups and was abnormal in IDWA and IDA was Ret-He, on which the diagnosis of iron deficiency was based.

In the whole study group (n = 90), there were only weak negative correlations between iron and Hepc, TSAT and Hepc, and a weak positive correlation between transferrin and Ret-He (Table 4).

In the subgroup of NIS patients, there was a moderate negative correlation between ferritin and Ret-He (r = −0.40, *p* = 0.008). In the IDWA subgroup, there was a moderate negative correlation between iron and Hepc (r = −0.51, *p* = 0.0005) and TSAT and Hepc (r = −0.44, *p* = 0.003).

**Figure 1:**
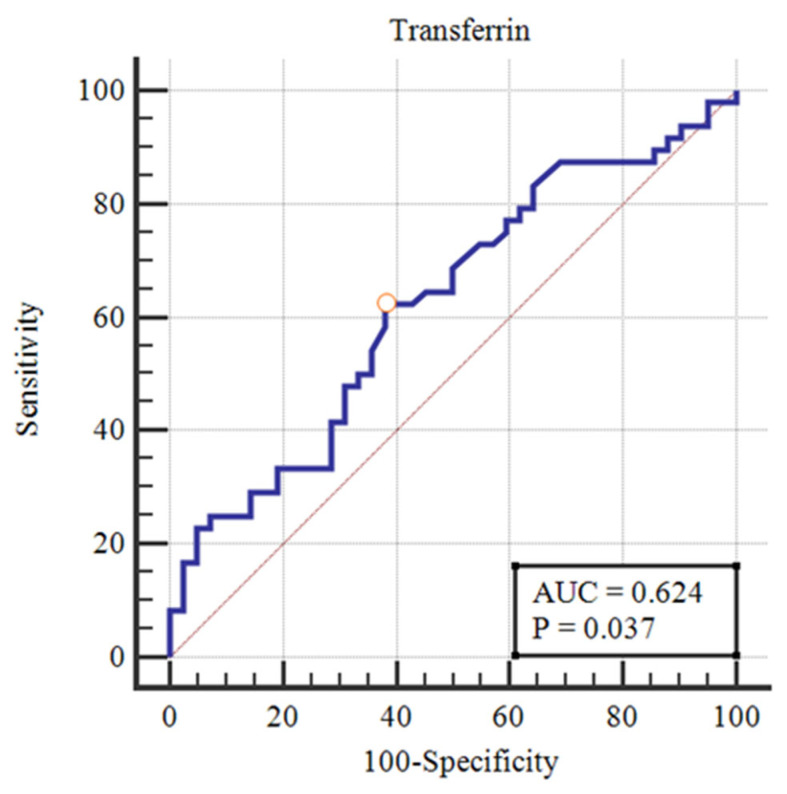
Receiver Operating Characteristic curve for transferrin concentration in the prediction of iron deficiency in sepsis patients.

The only standard iron biomarker that reached statistical significance for ID diagnosis was transferrin concentration with AUROC 0.62 (95%CI 0.52–0.72, *p* = 0.04), with the optimal cut-off value of ≤134 mg/dL (Figure 1); however, the AUC value is not high enough to consider transferrin to be a good clinical marker for differential diagnosis in ID. The other standard iron biomarkers in the prediction of ID did not reach statistical significance.

To determine additional differences in iron status markers, we performed calculations for between-group differences among study group patients divided into separate “sepsis” (n = 42/46.7%) and “septic shock” (n = 48/53.3%) subgroups. Only transferrin concentration varied between patients with sepsis (Me 141, IQR 117–186 mg/dL) versus patients with septic shock (Me 116, IQR 86–147 mg/dL); *p* = 0.008. Upon further investigation, the difference prevailed only in sepsis patients with IDA (n = 19; Me 134, IQR 112–162 mg/dL) versus septic shock patients with IDA (n = 23; Me 108, IQR 72–138 mg/dL); *p* = 0.019. Ferritin, TSAT, iron concentration, hepcidin, and Ret-He did not differ between sepsis and septic shock patients (*p* > 0.05 for all)

Furthermore, we performed correlation coefficient calculations for sepsis prognostic scores (APACHE II and SOFA) and iron markers. There were weak correlations for the SOFA score and ferritin (R = 0.36; *p* = 0.0005), TSAT (R = 0.26; *p* = 0.012), and iron concentration (R = 0.259; *p* = 0.014). There were also weak correlations for the APACHE II score and ferritin (R = 0.27; *p* = 0.01), TSAT (R = 0.28; *p* = 0.01), iron concentration (R = 0.27; *p* = 0.009), and Ret-He (R = −0.22; *p* = 0.04). 

## 4. Discussion

Our study attempted to compare standard iron tests with new laboratory biomarkers of iron status in sepsis patients across different iron status subgroups. Diagnosis of IDWA and IDA was based on Ret-He and Hb concentration. Ret Hb equivalent is a credible marker for determining the presence and severity of iron deficiency in different populations of patients: children [16] and adults [18]. Its validity has been reported [19,20].

Ganz et al. showed that ferritin and Hepc concentrations were significantly elevated in ICU patients, and their values were the highest in sepsis patients. Ferritin is a positive acute-phase protein with its concentration rising during systemic inflammation. On the contrary, serum iron and transferrin levels were decreased in ICU subjects, with the lowest values among sepsis patients [21]. Transferrin is a negative acute-phase protein whose concentration drops during systemic inflammation. This seems to agree with the results we acquired, confirming the presence of severe systemic inflammatory response in our study population. Another recent study by Boshuizen et al. explored iron metabolism in AI of critically ill patients hospitalized in the ICU [22]. The presented results showed an early decrease of iron, transferrin, and TSAT in AI patients upon the progression of the state of anemia, whereas ferritin and Hepc levels were increased in the AI population. In our study, we have observed a clear direction of changes in values of ferritin and transferrin levels. Other parameters under consideration, such as iron level, TSAT, and Hepc, had no evident trend in iron status subgroups in our study. Additionally, the absolute values did not differ enough between subgroups for these variables to act as markers of ID stages, varying only slightly in IDWA and IDA. 

Our study showed a moderate negative correlation between ferritin and Ret-He in sepsis patients with normal iron status (diagnosed as normal Ret-He). Davidkova et al. showed a negligible correlation between ferritin and Ret-He (r  =  0.09, *p*  =  0.04) in the general population of pediatric patients with end-stage chronic kidney disease. The subgroup analysis showed a weak correlation in hemodialysis patients (r  =  0.27, *p*  =  0.001) and no correlation in peritoneal dialysis patients (r  =  −0.03, *p*  =  0.63) [23]. The fact that correlations in the study by Davidkowa et al. and ours are opposite highlights that these two parameters do not correlate in patients with systemic inflammation (dialysis patients, sepsis patients). Only a weak correlation was shown by Davidkowa et al. between TSAT and Ret-He (r  =  0.34, *p*  <  0.001). The correlation coefficient between TSAT and Ret-He was moderate among hemodialysis patients (r  =  0.47, *p* < 0.001), whereas only a weak correlation was showed for TSAT and Ret-He in the subgroup of peritoneal dialysis patients (r  =  0.22, *p*  <  0.001) [23]. Our study found no correlation between these parameters in the whole group and the iron status subgroups. In the study of ICU patients, Zuther et al. analyzed the utility of reticulocyte parameters for the diagnosis of ID. The authors compared Ret-He and Delta-He (difference between Ret-He and RBC-He) with standard iron biomarkers. Correlations between standard iron biomarkers and Ret-He/Delta-He were primarily present in patients without ID and were weak, with the values of correlation coefficients varying between 0.199 to 0.347 (*p* < 0.05) [24]. Our study also showed a weak positive correlation between transferrin concentration and Ret-He (r = 0.24; *p* = 0.025). Only in the NIS group was there a moderate negative correlation between ferritin and Ret-He (r = −0.40, *p* = 0.008).

In the study by Ganz et al., the authors reported a moderate positive correlation between ferritin and Hepc in the general population of ICU patients (r = 0.58, *p* < 0.001). In our study, we showed no correlation between these two biomarkers.

A moderate negative correlation was found between iron, Hepc, and TSAT and Hepc in the IDWA sepsis patients [21]. Our results are similar to the results in the cohort of novel coronavirus disease (COVID-19) patients in whom the negative correlation between serum iron and Hepc was weak (r = −0.24, *p* = 0.04) [25]. This is quite striking as an infection is known to cause hypoferremia of inflammation, which in turn leads to anemia of inflammation [25]. The study by Frost et al. showed that an increased inflammatory response, resulting mainly from interleukin 6 influencing the increase of hepcidin concentration, should not be associated with low serum iron. Our results seem to follow those conclusions [25].

Several studies attempted to report the correlations between Hepc and other standard biomarkers for checking systemic iron status. Previously reported markers have certain limitations. For instance, starting with hemoglobin, which was discovered to have poor sensitivity and specificity for detecting iron deficiency [26]. Next, ferritin concentration in circulation is not exclusively related to iron storage capacity and has been rather linked to a ‘leakage’ from damaged cells [27]. In the case of the transferrin saturation, the established detection assays are not sensitive to small agitations in iron load and take the assumption that iron from other sources (e.g., non-transferrin bound iron and ferritin) is negligible [28]. Considering that Hepc expression directly influences all previously mentioned markers, high hopes were placed upon this marker as a potential indicator of iron stores, especially in assessing immediate iron needs. However, there are at least ten methods and assays for measuring Hepc. With discrepancies in the calculations, it is uncertain which assays are the most competent for setting the Hepc reference ranges and drawing comparisons with standard iron markers [29]. There are substantial variations between the assays in absolute Hepc values due to a lack of standard material, a standardized method, and a commutable calibrator [30]. In the study comparing mass (MS) spectrometry and immunochemical (IC) Hepc detection methods, concentrations of 0.0–44.4 nmol/L with MS and 0.08–155.2 nmol/L with IC were reported [30]. Furthermore, Hepc-to-transferrin saturation and Hepc-to-serum ferritin ratios should also be interpreted regarding age- and gender-specific values and reference ranges. Failing to unify the Hepc determination procedures may strongly impede further research toward the designation of the actual, credible correlations between this relatively new and other more classic iron metabolism biomarkers [31]. 

High TSAT, ferritin, serum iron, and decreased transferrin concentrations were associated with reduced survival among sepsis patients upon ICU admission in a study by Brandtner et al. performed in 2020. The authors linked the alterations in systemic iron homeostasis with patients’ outcomes in sepsis, showing positive correlations of increased serum iron and ferritin with the SOFA score, which is a staple in sepsis severity diagnostics [32]. 

Our results confirmed a negative correlation between ferritin and transferrin, although the coefficient achieved only moderate strength. Even though hemoglobin indicators and iron homeostasis parameters are not an element of the gradation of inflammatory response in the SOFA score itself [33], it may be of benefit to assess these biomarkers independently, adding them to the overall SOFA-based prognostication model calculated for sepsis patients upon ICU admission, particularly those considered to be iron deficient in any degree.

Ucar et al. evaluated the usefulness of Ret-He as a parameter that allows for the diagnosis of anemia and functional iron deficiency before it fully develops in a patient, and its increase can also be detected earlier than the increase of hemoglobin and a classical reticulocyte count, which is helpful in treatment evaluation and follow-up [34]. The authors found some meaningful correlations in the ID and IDA groups between the Ret-He and baseline ferritin, baseline iron level, transferrin saturation level, and hemoglobin, with the R-values ranging between 0.4 to 0.7 (*p* < 0.05 for all). However, the study subjects included hematology patients with no symptoms of infection, and the results obtained cannot be compared to those obtained in our study.

In the study among children with a high prevalence of infections (mainly malaria), Aguilar et al. analyzed the diagnostic accuracy of various biomarkers in the prediction of ID. The analyzed tests were compared with the diagnostic gold standard (bone marrow staining with the Perls’ Prussian blue). The AUROCs for ferritin, transferrin concentration, and TSAT were 0.70–0.71. In our study, the classic iron parameter that reached statistical significance was transferrin concentration with AUROC 0.62; however, with such an AUC value, we could not consider transferrin concentration to be a superior clinical parameter for ID diagnosis. The authors showed satisfactory AUROCS for soluble transferrin receptor (TfR) and transferrin-ferritin index (TfR-F index) in the prediction of ID, 0.75 and 0.76, respectively [35]. These two tests showed excellent diagnostic accuracy in the prediction of ID (confirmed with bone marrow staining) in patients with IDA and AI + IDA, with AUROCs of 0.98 and 1.00, respectively [36]. 

Our study has limitations. Firstly, it was a single-center study, therefore the results obtained would require external validation. However, it was performed as a prospective study, so the introduction of a potential bias was reduced. The number of study subjects is another limitation. Although, we screened all consecutive patients with sepsis or septic shock for 12 months. The group size was determined mainly by the fact that only sepsis patients with PCT > 0.5 ng/mL were enrolled in the study. Although the study group was small, it was numerous enough for statistical analysis to reach significance. Moreover, we performed an additional a posteriori power calculation regarding our project’s sample size for the study group to increase our results’ reliability. We discovered that we would require a minimum of 84 patients to verify the correlations between iron biomarkers in various iron status subgroups, with the value of correlation coefficient of at least R = 0.3 with an alpha of ≤0.05 and a beta of 0.20. Therefore, we acknowledge that our sample size is sufficient to reach conclusions and our study was not underpowered. Furthermore, the diagnosis of ID was based on Ret-He only. We did not use the gold-standard bone marrow staining method to confirm the diagnosis.

## 5. Conclusions

There are no meaningful correlations between standard and new iron biomarkers in various iron status subgroups of sepsis patients. Standard iron biomarkers cannot be used to diagnose iron deficiency in sepsis patients.

## Figures and Tables

**Table 1:** Diagnostic criteria for iron status subgroups.

Iron Status	Ret-He ^1^ [pg]	Hb ^2^ [g/L]
Normal	≥30.2	irrespective
Iron deficiency without anemia	<30.2	≥120/130
Iron deficiency anemia	<30.2	<120/130

^1^ Reticulocyte hemoglobin equivalent. ^2^ Hemoglobin.

**Table 2:** General laboratory parameters in the iron status subgroups (between-group differences calculated with the Kruskal–Wallis test for variables with non-normal distribution, values are medians with interquartile ranges; IQR).

Laboratory Parameter	NIS (n = 42)	IDWA (n = 6)	IDA (n = 42)	*p*	Reference Values
Procalcitonin [ng/mL]	2.88 (1.01–15.80)	1.97 (0.98–3.93)	5.59 (1.80–21.20)	0.27	<0.5
C-reactive protein [mg/L]	**121 (69–234)**	117 (59–238)	**239 (150–307)**	**0.002**	<0.5
Total bilirubin [mg/dL]	0.64 (0.34–1.09)	0.34 (0.30–0.49)	0.46 (0.29–1.10)	0.41	0.3–1.2
Alanine aminotransferase [u/L]	31.2 (19.1–84.9)	24.3 (10.6–126.0)	27.2 (18.4–89.0)	0.69	<35.0
Aspartate aminotransferase [u/L]	56.9 (31.2–108.0)	43.5 (27.1–184.0)	61.1 (30.8–132.0)	0.73	<35.0
Creatinine [mg/dL]	1.21 (0.70–1.99)	1.93 (1.55–4.03)	1.66 (0.76–2.55)	0.26	0.5–0.9
Blood urea nitrogen [mg/dL]	31.7 (20.8–45.3)	49.8 (45.2–52.3)	37.8 (24.7–47.4)	0.07	7.8–20.0
Red blood cells [10^6^/µL]	**3.58 (3.02–4.22)**	**4.66 (4.24–4.77)**	**3.16 (2.95–3.63)**	**<0.001**	4.50–5.90
Hematocrit [%]	**33.7 (29.4–38.9)**	**41.9 (40.1–43.0)**	**29.5 (26.1–31.7)**	**<0.001**	40–52
Mean cell volume [fL]	92.9 (88.5–96.9)	90.7 (78.4–99.8)	90.9 (85.6–95.5)	0.32	80–96
Mean cell hemoglobin [pg]	30.3 (29.3–31.7)	29.3 (25.2–30.3)	29.4 (27.8–30.4)	0.06	28–33
Mean cell hemoglobin concentration [g/dL]	32.6 (31.6–33.6)	32.1 (30.2–32.8)	32.1 (31.3–33.5)	0.35	33–36
Red blood cell distribution width (SD) [fL]	50.2 (45.8–55.0)	52.6 (48.5–56.5)	51.9 (45.1–55.8)	0.78	35.3–50.3
Red blood cell distribution width (CV) [%]	15.2 (14.0–16.8)	15.8 (14.4–16.9)	15.4 (14.0–17.2)	0.71	10–15

In bold statistically significant correlations verified by post-hoc analysis; NIS–normal iron status; IDWA—iron deficiency without anemia; IDA—iron deficiency anemia.

**Table 3:** Laboratory parameters of iron status in the study subgroups (between-group differences calculated with the Kruskal-Wallis test for variables with non-normal distribution, values are medians with interquartile ranges; IQR).

Laboratory Parameter	NIS (n = 42)	IDWA (n = 6)	IDA (n = 42)	*p*	Reference Values
Iron [µg/dL]	22.3 (14.3–38.5)	81.9 (22.2–141.0)	26.7 (13.6–42.4)	0.07	33–193
Transferrin [mg/dL]	141 (105–183)	138 (112–201)	120 (83–150)	0.07	200–360
Transferrin saturation [%]	12.7 (6.5–21.3)	42.1 (18.4–76.2)	15.9 (7.5–28.9)	0.05	20–40
Ferritin [ng/mL]	721 (313–1733)	570 (484–2215)	1059 (421–1641)	0.67	13–150/30–400
Reticulocytes [%]	**2.19** (**1.36–2.82**)	1.61 (0.67–2.30)	**1.44** (**1.10–2.03**)	**0.019**	0.5–1.5
Reticulocytes [106/µL]	**0.08** (**0.05–0.09**)	0.07 (0.03–0.09)	**0.05** (**0.04–0.06**)	**0.004**	0.05–0.12
Immature reticulocyte fraction [%]	16.9 (12.5–26.4)	22.0 (12.3–26.6)	18.2 (12.1–26.1)	0.85	1.0–8.9
High fluorescence reticulocytes [%]	5.0 (1.8–10.1)	5.4 (2.0–11.9)	3.5 (2.1–10.3)	0.97	0.0–6.3
Medium fluorescence reticulocytes [%]	12.3 (9.4–14.9)	15.3 (10.3–17.4)	13.1 (9.7–15.8)	0.46	2.6–13.8
Low fluorescence reticulocytes [%]	83.1 (73.6–87.5)	78.0 (73.4–87.7)	81.8 (73.9–87.9)	0.85	84.4–96.5
Reticulocyte hemoglobin equivalent [pg]	**33.1** (**31.4–34.8**)	**28.5** (**23.1–29.9**)	**27.0** (**24.0–28.8**)	**<0.0001**	30.2–36.2
Hemoglobin [g/L]	**109** (**92–124**)	**137** (**121–140**)	**93** (**83–101**)	**<0.001**	140–175
Hepcidin [pg/mL]	1184 (664–2462)	1463 (762–2498)	751 (413–2287)	0.13	undetermined

In bold statistically significant correlations verified by post-hoc analysis; NIS—normal iron status; IDWA—iron deficiency without anemia; IDA—iron deficiency anemia.

**Table 4:** Correlation coefficients between standard and new iron biomarkers in the study group (calculated with Spearman’s rho correlation coefficient for variables with non-normal distribution, values are R-correlation coefficients with the corresponding *p*-value).

Parameter	Iron	Transferrin	Transferrin Sat.	Ferritin	Hepcidin	Ret-He
Iron [µg/dL]	-	R = −0.02; *p* = 0.85	**R = 0.88; *p* < 0.0001**	**R = 0.39; *p* < 0.0001**	**R = −0.26; *p* = 0.014**	R = −0.03; *p* = 0.81
Transferrin [mg/dL]		-	**R = −0.45; *p* < 0.0001**	**R = −0.48; *p* < 0.0001**	R = −0.05; *p* = 0.64	**R = 0.24; *p* = 0.025**
Transferrin sat. [%]			-	**R = 0.53; *p* < 0.0001**	**R = −0.22; *p* = 0.041**	R = −0.12; *p* = 0.25
Ferritin [ng/mL]				-	R = −0.02; *p* = 0.84	R = −0.18; *p* = 0.09
Hepcidin [pg/mL]					-	R = 0.17; *p* = 0.12
Ret-He [pg]						-

In bold statistically significant correlations; Transferrin sat.—transferrin saturation; Ret-He—reticulocyte hemoglobin equivalent.

## Data Availability

The data presented in this study are available on request from the corresponding author.
